# 

**Published:** 1925

**Authors:** 


					THE
BRISTOL
MEDICO-CHIRURGICAL
JOURNAL
PUBLISHED UNDKR TBS AUSPICES OT
THE BRISTOL MEDICO-CHIRURGICAL SOCIETY.
0pENlNG OF NEW UNIVERSITY BUILDINGS
BY
1b.flD. Gbe Iking.
(W.
J(JflS2ig 25
v^/// *\>
Ionian 0e^?>
Vol. XLII. No. 156.
"Contents" inside cover.

				

